# Biomechanical evaluation of screw and cement placement strategies for treating medial uncontained tibial defects in total knee arthroplasty: A finite element analysis

**DOI:** 10.3892/mi.2024.171

**Published:** 2024-06-25

**Authors:** Atichart Kwanyuang, Phachara Suklim, Khanin Iamthanaporn, Varah Yuenyongviwat

**Affiliations:** 1 Institute of Biomedical Engineering, Faculty of Medicine, Prince of Songkla University, Songkhla 90110, Thailand; 2 Department of Orthopedics, Faculty of Medicine, Prince of Songkla University, Songkhla 90110, Thailand

**Keywords:** total knee arthroplasty, bone defect, screw augmen-tation, bone cement

## Abstract

Total knee arthroplasty faces challenges in the management of medial uncontained tibial defects, affecting prosthesis stability and implant survival. The use of screws and bone cement is a preferred approach; however, optimal screw insertion techniques lack consensus in the existing literature. The present study aimed to address this gap by exploring optimal screw and cement placement strategies, focusing on their biomechanical implications. The present study conducted a finite element analysis using a knee prosthesis model with a defined uncontained tibial defect. Various parameters were systematically adjusted, including the number of screws (1, 2 or 3 screws), screw lengths (10, 18, 30 or 40 mm), lateral-medial screw positions (2, 4 or 6 mm laterally) and abduction rotation angles (0, 5, 10 or 15 degrees). These adjustments were made to evaluate their specific and combined impacts on the vertical displacement and abduction angles of the tibial tray. The results revealed that incorporating three-screw reinforcement markedly reduced vertical displacement, while the single screw in the middle position exhibited superior performance in preventing the deformation of abduction angles compared to scenarios with two screws at anterior and posterior positions without a middle screw. Longer screws and smaller abduction angles contributed to decreased movement of the tibial component. Furthermore, the lateral adjustment of the screw position led to an increase in vertical displacement values, reaching ~1.5% when shifted 6 mm laterally. On the whole, the finite element analysis in the present study suggests that, for the treatment of medial uncontained tibial defects, three-screw reinforcement is advantageous for larger defects. Longer screws and a smaller abduction angle are deemed favorable. Moreover, the results underscore the superiority of medial screw placement over lateral placement. It is imperative to note that further clinical validation is essential to corroborate the biomechanical implications observed herein.

## Introduction

Knee arthroplasty is a valuable surgical intervention that markedly enhances the quality of life of patients suffering from knee joint degeneration. This procedure is considered for patients with varying degrees of disease severity, aiming to alleviate pain and restore joint function (1). However, in some cases, patients present with pre-existing bone loss in the tibia area, leading to more complex surgical challenges (2,3).

The successful correction of bone loss in the tibia region depends on the type and extent of the bone defect. The primary objective of correcting a bone defect is to establish a stable foundation capable of adequately supporting the knee joint components, ensuring the weight-bearing capacity of the femoral and tibial components without compromise (4,5). This, in turn, enables patients to regain mobility and engage in weight-bearing activities more efficiently post-surgery. Several methods are employed to address bone defects, including the use of bone cement (6), bone grafting (7), metal and rods (8) and combinations thereof (9).

For the treatment of uncontained or segmental defects, which entail the loss of bone extending from the internal bone tissue to the outer edge, the combination of screws and bone cement has emerged as the preferred approach (10). The screws play a crucial role in reinforcing bone cement by providing an anchor for it to adhere to, thereby increasing the overall stability (11). This approach is particularly suitable for cases where the bone defect comprises <50% of the tibial plateau on each side and the defect is <1 cm in depth. Studies with long-term follow-ups have reported positive outcomes using this method (12-14).

Despite the effectiveness of the screw-cement combination, there is currently a wide array of screw types available. The choice of which to use remains largely dependent on individual surgeon preference and familiarity. Notably, there exists a limitation in comparative studies evaluating the clinical efficacy and mechanical impact of various screw insertion factors, such as the screws themselves or the methods by which they are anchored to the bone. Consequently, there is a need to investigate these factors, which, when utilized in total knee arthroplasty (TKA) for treating limited marginal tibial bone loss, may yield distinct effects.

The present study aimed to address this gap by employing finite element analysis, an advanced computer technique for structural stress analysis developed in engineering mechanics. The aim was to evaluate the mechanical effects of using a different number of screws, screw locations, screw lengths and angles of screw insertion. The findings presented herein may provide valuable insight for surgeons when selecting the most appropriate screw conditions for their surgical interventions.

## Materials and methods

The present study commenced with the construction of a knee prosthesis model featuring an uncontained bone defect, specifically constituting 50% of the medial tibial plateau. The dimensions of the defect are precise, measuring 1 cm in depth and an incline at 30 degrees. Various parameters were systematically adjusted in the computer model and evaluated by finite element analysis to assess their impact on the model. This involved testing different quantities of screws (1, 2 or 3 screws), various screw lengths (10, 18, 30 or 40 mm), and exploring different screw positions with lateral shifts of 2, 4 or 6 mm from the original sites. Additionally, rotational adjustments were examined, introducing angles of 5, 10 and 15 degrees away from the original sites in an abduction direction.

### Finite element model of knee arthroplasty with an uncontained tibial bone defect model construction

An artificial left tibia (Sawbones^®^ #3401 4th gen., Composite, 17 PCF Solid Foam Core, Medium, Pacific Research Laboratories, Inc.) with a tibial tray (Zimmer^®^ NexGen^®^ Complete Knee Solution, Size 5, Zimmer Biomet Holdings, Inc.), along with relevant components including polymethylmethacrylate (PMMA) bone cement and Ø3.5 mm screws, was meticulously assembled to replicate the conditions of knee arthroplasty with an uncontained tibial bone defect. This construct was transformed into a three-dimensional (3D) model by utilizing a series of 256 computed tomography (CT) images, each boasting a resolution of 512x512 pixels and a slice thickness of 1.0 mm. The processes involved in creating the 3D model of knee arthroplasty with an uncontained tibial bone defect encompassed the segmentation of artificial bone and implant regions, followed by voxel generation for constructing the 3D model. These steps were executed using Mimics Research software (version 20.0, Materialise NV).

Subsequently, the 3D model, with a particular focus on the tibial cortical and cancellous bones, underwent enhancement using 3-matic research software (version 12.0, Materialise NV). This enhancement process included the removal of extraneous components, rectification of defects and the refinement of surfaces to attain a precise representation of the intended construct.

To finalize the 3D model of knee arthroplasty with an uncontained tibial bone defect, the enhanced 3D models of the construct were imported into NX software (version 12.0, Siemens Product Lifecycle Management Software, Inc.) for further modifications, employing a range of functions available within the software. The unnecessary distal portion of the artificial tibia was removed to economize the analysis, and the uncontained bone defect of 50% on the medial tibial plateau was refined. Due to the metal-related artifacts evident in the CT images, it was imperative to accurately reproduce the tibial tray and the screws. The geometry of the tibial tray was simplified, while the geometry of screw was enhanced to adhere to the ISO standard for metallic bone screws. A layer of PMMA bone cement was modeled to correspond with the surrounding tibial tray and tibial bone. The aim was to ensure adhesion between the tibial tray and tibial bone, as well as to fill the existing defect. However, the original positions of all 3D components were maintained in alignment with the CT images.

**Figure 1:**
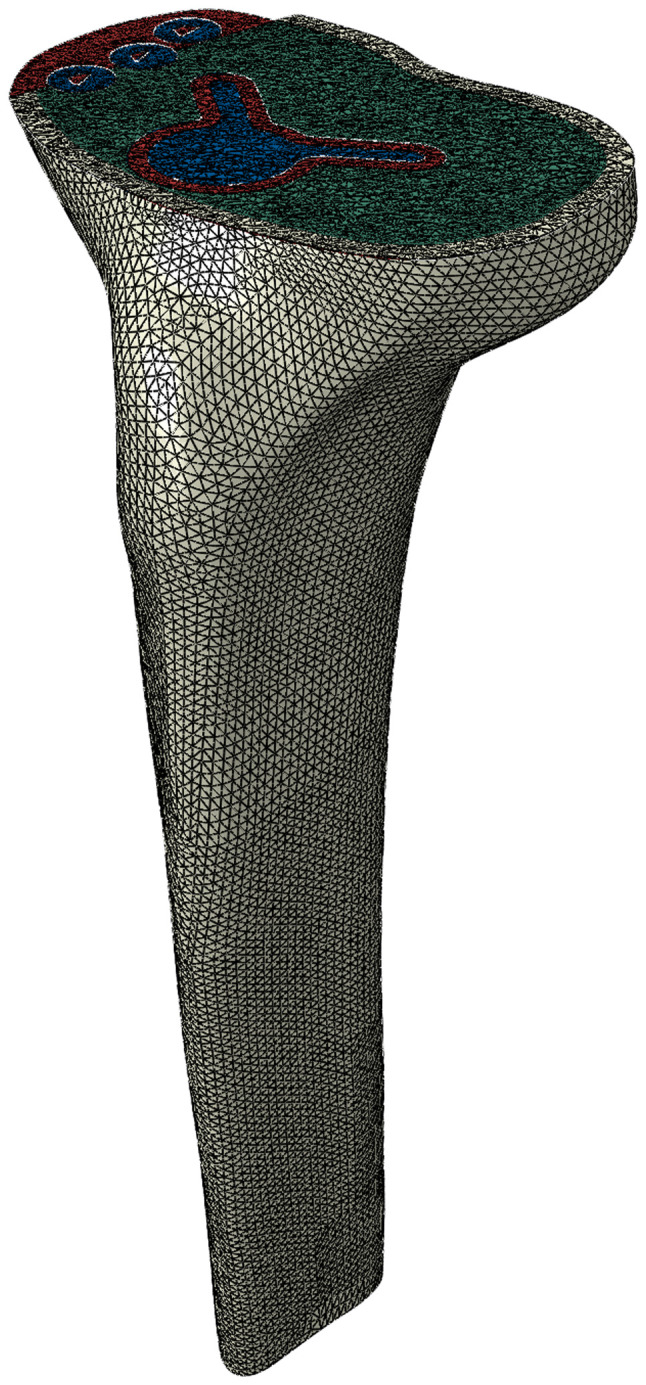
Three dimensional model of the construct.

The completed 3D model of the construct was then imported into Abaqus/CAE software (version 2020, Dassault Systèmes Simulia Corp.) to commence the creation of a finite element model. At this stage, the tibial cancellous bone was interconnected with the tibial cortical bone, and cavities were simulated to mimic the fixing hole of the screw and the installation hole for the stem of the tibial tray in relation to the PMMA bone cement and the tibial bone (Fig. 1).

### Material properties

The material properties of all components in the present study were assumed to be homogenous, isotropic and linearly elastic. The Young's moduli of the synthetic cortical and cancellous bones, as provided by the manufacturer, were 16.7 GPa and 155 MPa respectively, with Poisson's ratios of 0.30 for both. The tibial tray and all screw types were assumed to be composed of a titanium alloy, possessing Young's modulus and Poisson's ratios of 110 GPa and 0.3, respectively (15). The corresponding values for the PMMA bone cement were 2.28 GPa for the Young's modulus and 0.3 for the Poisson's ratio (16).

### Interactions, loads and boundary conditions

In the present study, the PMMA bone cement served as the adhesive that secured all components, including the tibial bone, bone screw and tibial tray. These interrelated properties were established as fully constrained to simulate the adhesive force of the PMMA bone cement. However, the distal part of the bone screw extended beyond the boundary of the PMMA bone cement and penetrated into the tibial bone. Defining the bone-screw interface property was also imperative. The contact properties between the tibial bone, encompassing both cortical and cancellous sections, and the bone screws were categorized into two types. The interface of the bone screw was assigned a coefficient of friction of 0.3.

The distal end of the tibial shaft was entirely immobilized. A compressive force of 2,100 N, equating to 2-3-fold the normal body weight, was exerted on the tibial tray through two loading points; one loading point was positioned approximately at the midpoint of the medial tibial plateau, while the other was situated near the midpoint of the lateral tibial plateau. Furthermore, the loading conditions were bifurcated, 55 and 45% of the total load were applied to the medial and lateral tibial plateaus, respectively, emulating normal varus-valgus alignment.

A mesh convergence study and sensitivity analysis were conducted on models representing the tibial bone, as well as bone screws. These analyses were performed to enhance the accuracy and reliability of the simulation results.

## Results

### Finite element analysis of different quantities of screws (Fig. 2)

**Figure 3:**
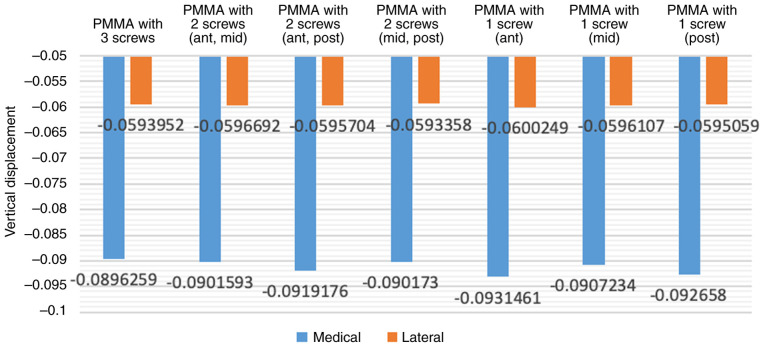
Vertical displacement of tibial component with varying numbers and positions of screws.

The comparison of the effects of the three-screw reinforcement with those of one- or two-screw reinforcement in the uncontained tibial bone defect filled with bone cement model revealed that the vertical displacement values in the three-screw reinforcement condition were lower (Fig. 3). However, in specific positions, a single screw exhibited inferior load-bearing performance when compared to certain scenarios involving the use of two screws. Notably, the utilization of a single screw in the middle position exhibited superior performance in preventing deformation in the form of abduction angles compared to the use of two screws at anterior and posterior positions without a middle screw. Placing a screw in the middle position appears to play a crucial role in enhancing load-bearing capacity.

### Finite element analysis of different lengths of screws

**Figure 4:**
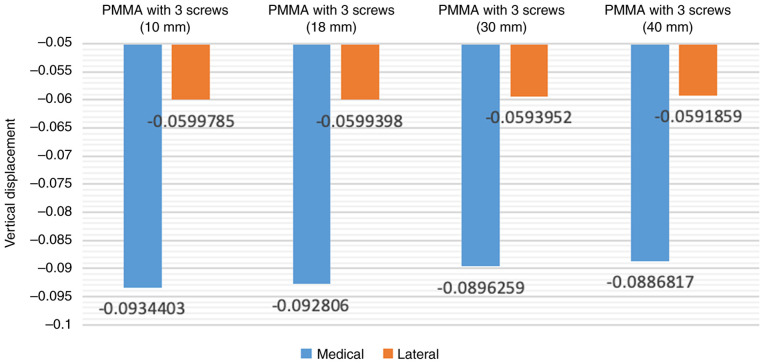
Vertical displacement of tibial component with varying screw lengths.

In the initial model, the screw length employed in the present study was 30 mm. Increasing the screw length to 40 mm resulted in a reduction of vertical displacement values by up to 1%, and the abduction angle decreased by approximately 2.5%. Conversely, the vertical displacement values measured at the loading point on the tibial tray increased when the screw lengths were decreased (10 and 18 mm screws), exhibiting an inverse variation. The findings indicated that the length of screw reinforcement had a direct association with the load-bearing performance of tibial arthroplasty in the condition of an uncontained tibial bone defect filled with bone cement (Fig. 4).

### Finite element analysis of screw placement locations

**Figure 5:**
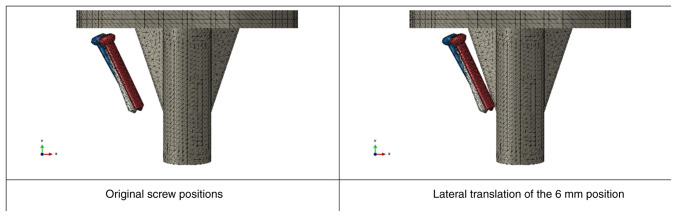
Screw placement locations.

**Figure 6:**
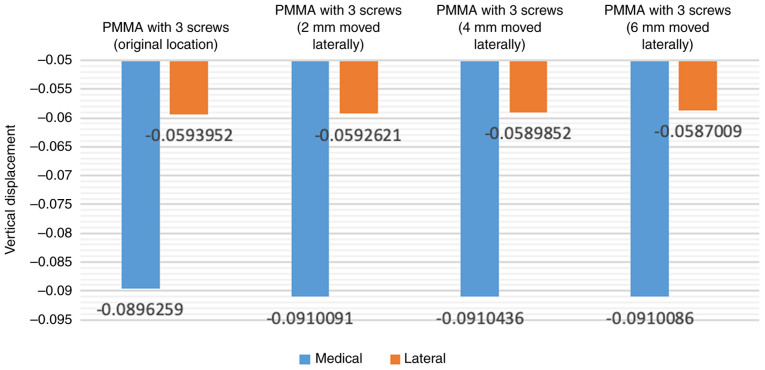
Vertical displacement of tibial component with different screw placement locations.

When adjusting the position of the screw laterally to gradually approach the center of the tibial tray (2, 4 and 6 mm laterally), there was an increase in vertical displacement values (Fig. 5). Shifting the screws laterally to increase the distance from the original location of the screw to 6 mm resulted in an increase of ~1.5% in vertical displacement values and around 6.9% in the abduction angle (Fig. 6).

**Figure 7:**
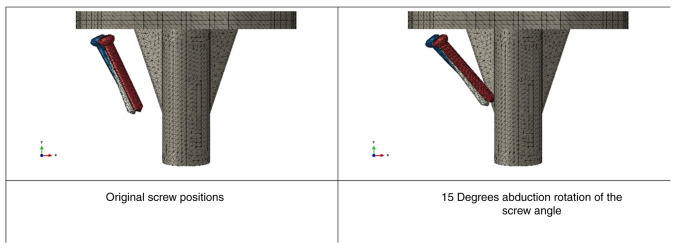
Screw placement angle.

**Figure 8:**
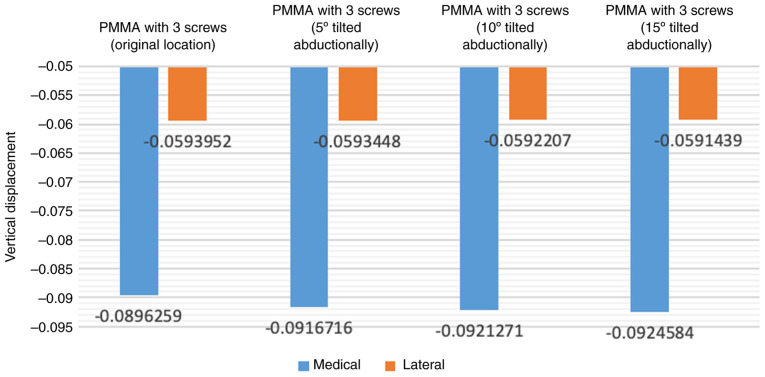
Vertical displacement of tibial component with varying screw angle.

Similarly, when incrementally increasing the degree of abduction rotation of the screw angle (5, 10 and 15 degrees), the load-bearing performance gradually diminished (Fig. 7). Rotating the screws further in abduction to increase the angle from the original location of the screw to 15 degrees led to an increase of ~3.2% in vertical displacement values and ~10.2% in the abduction angle (Fig. 8).

## Discussion

Bone defects pose a significant challenge in TKA as they affect prosthesis stability and may affect implant survival (17,18). The combination of screws and bone cement has been identified as an effective strategy to address this issue, as evidenced by the successful long-term results reported by Ritter and Harty (19). In their study involving 125 TKA with metal-backed tibial components, screws in cement were employed to manage medial defects caused by large varus deformities. With an average follow-up of 7.9 years, only two knees (1.6%) exhibited medial collapse, with no need for revision. Özcan *et al* (10) also reported positive outcomes in high body mass index patients, where no implant failure or revision was observed during the follow-up period. However, the existing literature lacks consensus on optimal screw insertion techniques. The aim of the present study was to evaluate the mechanical effects of different screw parameters, including the number of screws, screw positioning, length and angulation, in uncontained medial tibial defects using finite element analysis. The results suggest that three-screw reinforcement is preferable for larger defects, the middle position is crucial for enhancing load-bearing capacity, longer screws are advantageous, a smaller abduction angle is favorable and medial placement outperforms lateral placement.

In the present study, the three-screw reinforcement condition demonstrated the lowest displacement values, aligning with the findings of a prior study conducted by Zheng *et al* (20). Zheng *et al* (20) assessed the impact of the number of screws on stresses at the surface of uncontained tibial defects using finite element analysis and reported that in models with a 12% defect area and a 12-mm depth, the use of two screws, as opposed to a single screw, led to a reduction in stresses on the defect surface. Furthermore, a separate finite element study by Zhao *et al* (21) revealed that in models with an 18% defect area and a 15 mm depth, higher bone stresses were observed with the use of one screw compared to two screws. Despite variations in the specific metrics measured between studies, the collective evidence emphasizes the beneficial effects of increasing the number of screws in reinforcing tibial defects. This augmentation may contribute to an overall improvement in stability within the context of tibial prostheses.

The present study identified a notable reduction in vertical displacement of the tibial tray with the use of longer screws. This observation contrasts with the findings of a prior orthopedic study by Ma *et al* (22), employing finite element analysis to evaluate the impact of screw length in tibial defects during TKA with a cement-screw technique. In the study by Ma *et al* (22), contact stresses on the surface of cancellous bone screws remained consistent, exhibiting no significant difference with varying screw lengths of the same diameter. Additionally, stresses on the tibial surface did not exhibit notable variations based on screw length. It is suggested that the discrepancy in outcomes may be attributed to differences in defect size. The study by Ma *et al* (22) focused on smaller defects, comprising <12% of the total plateau and with a depth of <10 mm. By contrast, the present study specifically addressed larger defects, constituting ~25% of the total plateau. This substantial difference in defect size may underlie the observed variations in the influence of different screw lengths on vertical displacement in the present study, highlighting the clinical relevance of defect dimensions in TKA outcomes. However, the authors still believe that in the clinical setting, choosing longer screws may provide a benefit without incurring additional costs or risks for the patient. Future comparative studies evaluating different screw lengths in clinical settings would be beneficial to further guide optimal screw selection for patient outcomes.

The present study observed a gradual diminishment in load-bearing performance with decreasing vertical angles of the screw. This trend concurs with the findings of a pertinent finite element study conducted by Zheng *et al* (20). In their study conducted on models with a 12% defect area and a 12-mm depth, the researchers reported that utilizing one vertical screw for defect reconstruction resulted in a lower focused stress within the cancellous bone surrounding the screws (1.05 MPa) compared to the stress observed with one oblique screw (1.23 MPa). Additionally, a complementary finite element study by Zhao *et al* (21), examining models with a 9% defect area and an 8 mm depth, demonstrated that the use of one vertical screw yielded significantly lower focused stress (0.45 MPa) than one oblique screw (1.72 MPa). These consistent findings underscore the clinical importance of screw angles in influencing focused stress within the cancellous bone, emphasizing its significance for optimizing load-bearing performance, as reported in orthopedic literature.

The present study has a number of limitations that warrant consideration. Firstly, the finite element analysis relies on simulation, offering controlled experimentation, but potentially deviating from the intricate *in vivo* conditions. Secondly, the study specifically targeted a defect size of 25% of the total plateau, and caution should be exercised in applying the findings to cases with different defect dimensions. Third, the assumption of material homogeneity may not fully capture clinical variations and did not account for potential individual variations in bone quality, factors that could have implications. Finally, the analysis, centered on mechanical aspects, necessitates further validation through experimental and clinical studies for robust clinical translation. Acknowledging these limitations is crucial for a nuanced interpretation of the outcomes of the study and offers directions for future research to systematically address these constraints.

In conclusion, the finite element analysis performed in the present study suggested that, for the treatment of medial uncontained tibial defects, three-screw reinforcement is advantageous for larger defects. Longer screws and a smaller abduction angle are deemed favorable. Moreover, the results underscore the superiority of medial screw placement over lateral placement. It is imperative to note that further clinical validation is essential to corroborate the biomechanical implications observed herein.

## Figures and Tables

**Figure 2:**
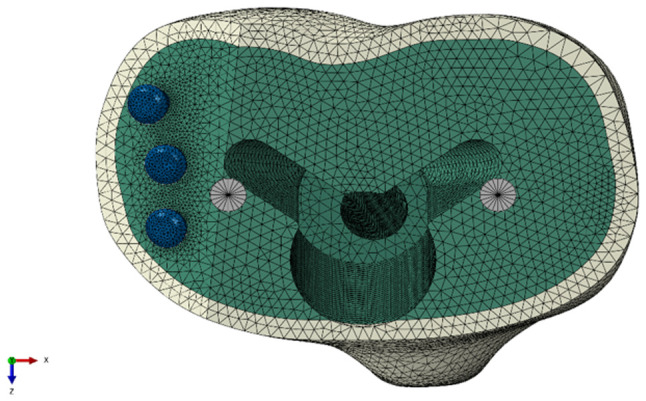
The position of the screws.

## Data Availability

The datasets used and/or analyzed during the current study are available from the corresponding author on reasonable request.

## References

[1] Kim SE, Ro DH, Lee MC, Cholewa JM (2023). Early- to mid-term review of a prospective, multi-center, international, outcomes study of an anatomically designed implant with posterior-stabilized bearing in total knee arthroplasty. Medicina (Kaunas).

[2] Alasaad H, Ibrahim J (2023). Primary total knee arthroplasty in patients with a significant bone defect in the medial tibial plateau: Case series and literature review. Int J Surg Case Rep.

[3] Lei PF, Hu RY, Hu YH (2019). Bone defects in revision total knee arthroplasty and management. Orthop Surg.

[4] Tang Q, Guo S, Deng W, Zhou Y (2023). Using novel porous metal pillars for tibial bone defects in primary total knee arthroplasty. BMC Musculoskelet Disord.

[5] Brooks PJ, Walker PS, Scott RD (1984). Tibial component fixation in deficient tibial bone stock. Clin Orthop Relat Res.

[6] Engh GA, Ammeen DJ (1999). Bone loss with revision total knee arthroplasty: Defect classification and alternatives for reconstruction. Instr Course Lect.

[7] Cuckler JM (2004). Bone loss in total knee arthroplasty: Graft augment and options. J Arthroplasty.

[8] Rand JA (1991). Bone deficiency in total knee arthroplasty. Use of metal wedge augmentation. Clin Orthop Relat Res.

[9] Whittaker JP, Dharmarajan R, Toms AD (2008). The management of bone loss in revision total knee replacement. J Bone Joint Surg Br.

[10] Özcan Ö, Yeşil M, Yüzügüldü U, Kaya F (2021). Bone cement with screw augmentation technique for the management of moderate tibial bone defects in primary knee arthroplasty patients with high body mass index. Jt Dis Relat Surg.

[11] Ritter MA (1986). Screw and cement fixation of large defects in total knee arthroplasty. J Arthroplasty.

[12] Ritter MA, Keating EM, Faris PM (1993). Screw and cement fixation of large defects in total knee arthroplasty. A sequel. J Arthroplasty.

[13] Berend ME, Ritter MA, Keating EM, Jackson MD, Davis KE (2014). Use of screws and cement in primary TKA with up to 20 years follow-up. J Arthroplasty.

[14] Cinotti G, Perfetti F, Petitti P, Giannicola G (2022). Primary complex total knee arthroplasty with severe varus deformity and large bone defects: Mid-term results of a consecutive series treated with primary implants. Eur J Orthop Surg Traumatol.

[15] Completo A, Rego A, Fonseca F, Ramos A, Relvas C, Simões JA (2010). Biomechanical evaluation of proximal tibia behaviour with the use of femoral stems in revision TKA: An in vitro and finite element analysis. Clin Biomech (Bristol, Avon).

[16] Zhao Y, Robson Brown KA, Jin ZM, Wilcox RK (2012). Trabecular level analysis of bone cement augmentation: A comparative experimental and finite element study. Ann Biomed Eng.

[17] Qiu YY, Yan CH, Chiu KY, Ng FY (2012). Review article: Treatments for bone loss in revision total knee arthroplasty. J Orthop Surg (Hong Kong).

[18] Mancuso F, Beltrame A, Colombo E, Miani E, Bassini F (2017). Management of metaphyseal bone loss in revision knee arthroplasty. Acta Biomed.

[19] Ritter MA, Harty LD (2004). Medial screws and cement: A possible mechanical augmentation in total knee arthroplasty. J Arthroplasty.

[20] Zheng C, Ma HY, Du YQ, Sun JY, Luo JW, Qu DB, Zhou YG (2020). Finite element assessment of the screw and cement technique in total knee arthroplasty. Biomed Res Int.

[21] Zhao G, Yao S, Ma J, Wang J (2022). The optimal angle of screw for using cement-screw technique to repair tibial defect in total knee arthroplasty: A finite element analysis. J Orthop Surg Res.

[22] Ma J, Xu C, Zhao G, Xiao L, Wang J (2023). The optimal size of screw for using cement-screw technique to repair tibial defect in total knee arthroplasty: A finite element analysis. Heliyon.

